# Immunological Analysis of a CCHFV mRNA Vaccine Candidate in Mouse Models

**DOI:** 10.3390/vaccines7030115

**Published:** 2019-09-16

**Authors:** Touraj Aligholipour Farzani, Katalin Földes, Koray Ergünay, Hakan Gurdal, Aliye Bastug, Aykut Ozkul

**Affiliations:** 1 Virology Department, Faculty of Veterinary Medicine, Ankara University, 06110 Ankara, Turkey; touraj.farzani@gmail.com (T.A.F.); fldeskatalin@gmail.com (K.F.); 2 Virology Unit, Department of Medical Microbiology, Faculty of Medicine, Hacettepe University, 06100 Ankara, Turkey; ekoray@hacettepe.edu.tr; 3 Pharmacology Department, Faculty of Medicine, Ankara University, 06100 Ankara, Turkey; Hakan.Gurdal@medicine.ankara.edu.tr; 4 Infectious Disease Department, Ankara Numune Training and Research Hospital, 06800 Ankara, Turkey; dr.aliye@yahoo.com; 5 Biotechnology Institute, Ankara University, 06560 Ankara, Turkey

**Keywords:** conventional naked mRNA, CCHFV, IFNα/β/γR^−/−^, specific immune responses

## Abstract

Development of new vaccine platforms against viral diseases is considered urgent. In recent years, mRNA constructs have attracted great interest in this field due to unique advantages over conventional gene transfer platforms. In the present study, we developed a new naked conventional mRNA vaccine expressing the non-optimized small (S) segment of the Ank-2 strain of Crimean-Congo Hemorrhagic Fever virus (CCHFV). We then analyzed its single and booster dose immunogenicity and protection potential in the challenge assay in two mice models, including IFNα/β/γR^−/−^ and C57BL/6. The results obtained from the immunological assays, namely IL-4 and IFN-gamma ELISPOT, intracellular IFN-gamma staining, in-house sandwich ELISA, and survival data, demonstrated that our construct elicited the production of anti-nucleocapsid (N) specific immune responses in both mice models. A 100% protection rate was only obtained in the booster dose group of IFNα/β/γR^−/−^ mice, indicating that this platform needs further optimization in future studies. In conclusion, we assessed a novel approach in CCHFV vaccination by introducing a conventional mRNA platform which can be considered in future experiments as an efficient and safe way to battle this disease.

## 1. Introduction

Crimean-Congo Hemorrhagic Fever (CCHF) is considered to be one of the main health concerns in endemic areas, from western China across southern Asia to the Middle East, Spain, and the Balkans, and throughout most of Africa [1].

The causative agent belongs to the *Bunyaviridae* family, which includes a tripartite RNA genome of negative polarity [2]. Due to wide geographical distribution, lack of a licensed vaccine or therapeutics, and high mortality rate, efficient vaccine development is a challenging issue in endemic areas like Turkey. Although protection strategies can reduce the mortality rate of human cases, it is important to design effective vaccines to target the disease in animals to eliminate zoonosis risk [3].

The main strategies for designing a vaccine against CCHFV include targeting the small (S) and medium (M) segments. However, due to uncontrolled mutations in the M segment (69–99% amino acid identity among strains), especially in mature glycoprotein precursor (GP), some researchers have focused on the S segment (91–99% amino acid identity among strains) as an alternative approach [4]. T and B cell epitopes of the S segment have not been fully characterized. Additionally, it has been documented that antibodies produced against this protein have no neutralizing effect on the virus. However, despite the lack of neutralizing antibodies, vaccination using different expression platforms delivering the S segment sufficiently stimulated the immune response to protect the challenged mice models [5,6,7,8]. To clarify this phenomenon, one study documented that vaccines based on this gene elicited balanced Th1 and Th2 responses, which resulted in protection in genetically modified mice models against lethal doses [9]. 

Nowadays, researchers mainly focus on designing new effective vaccine platforms to deliver immunodominant genes (S or M) of CCHFV with minimum drawbacks of well-known expression platforms. Our group has obtained 100% protection rate in the challenged IFNα/β/γR^−/−^ mice, using our newly developed Bovine herpesvirus 4-based viral vector that expresses the anti-nucleocapsid protein [8]. In another recent study, a vesicular stomatitis virus-based vaccine delivering the CCHFV glycoprotein precursor (GPC) protected STAT-1 knock-out mice against CCHF [10]. Recently, a single-dose replicon particle vaccine platform based on the reverse genetics system provided complete protection against CCHFV in IFNAR−/− mice [11].

Messenger RNA constructs are effective methods of gene delivery for vaccination due to various characteristics [12]. Specifically, this expression platform is safe and well tolerated, easy to propagate by commercially available kits at a satisfactory pure injectable level, and can be stabilized at room temperature by performing some modifications in its structure. Additionally, mRNA-mediated gene transfer occurs in non-dividing cells, which offers the advantage of not being restricted to a subject-specific human leukocyte antigen (HLA) allele [13].

Messenger RNA vaccines are mainly classified into two main categories: non-replicating, conventional mRNA; and virally derived, self-amplifying RNAs. One of the main considerations in designing a conventional mRNA vaccine is translation optimization, which can affect stability during in vivo experiments. This can be achieved by adding features to the mRNA structure during its synthesis such as 5′ and 3′ untranslated regions (UTRs), a 5′ cap structure (it can be easily added by a vaccinia virus capping enzyme [14], incorporating a synthetic cap [15], or anti-reverse cap analogs [16]), polyA tail, and codon optimization to avoid rare amino acids with low utilization [17].

As previously demonstrated, inherited immunostimulation of exogenous mRNAs may help gene transfer trials in some cases by providing adjuvant activity to drive dendritic cell (DC) maturation, which then elicits robust T and B cell immune responses [18]. This mechanism originates from their recognition by diverse cell surfaces, and endosomal and cytosolic innate immune receptors, such as toll-like receptors (TLRs) [19,20].

Various studies have demonstrated the potential of the mRNA constructs as an efficient and safe platform for human trials [21]. Recent studies have shown that a mix of three mRNA molecules encoding the co-stimulatory molecule CD70 and two dendritic cells (DC) activation stimuli, the CD40 ligand, and active TLR4 (TriMix) are well-tolerated and provide satisfactory outcomes in stage III/IV melanoma patients [22]. In another study, a phase I clinical trial of an intranodal administered mRNA-based therapeutic vaccine against HIV-1 infection elicited moderate HIV-specific T-cell responses in 21 chronic HIV-1-infected patients undergoing antiretroviral therapy (ART) [23].

To introduce a new expression platform in the struggle against this disease, we developed a conventional naked mRNA-based construct expressing the non-optimized S segment of the Ank-2 strain of CCHFV to evaluate its immunogenicity and protection against a challenge assay in two different mice models, namely C57BL/6 and IFNα/β/γR^−/−^. In the present study, firstly, we aimed to ascertain that small segment has potential to sufficiently elicit immune responses, especially, in the challenge assay; and secondly, we evaluated the efficacy of the conventional naked mRNA platform in battling against the CCHFV.

## 2. Materials and Methods

### 2.1. Ethics Statement

This study was carried out in strict accordance with the recommendations listed in the Guide for the Care and Use of Laboratory Animals of the Ankara University Ethical Committee for Animal Experiments (17 December 2014; 2014-23-155 and 17 October 2018; 2018-20-130). To provide humane endpoints during the experiments, free access to sterilized water and food were provided. At the end of each experiment, mice were euthanized by CO_2_ inhalation and cervical dislocation. C57BL/6 and Interferon alpha/beta/gamma receptor-null mice (IFNα/β/γR−/−; AG129; Strain: 129S7/SvEvBrd) were purchased from B&K Universal Ltd (Marshall Bioresouces, Hull, East Yorkshire, UK) and housed in the pathogen-free animal bio-safety level 3 plus (ABSL3+) facilities of the Virology Department, Veterinary Faculty of Ankara University, Turkey. All biological assays, including virus cultivation and virus preparation for injection, were also performed in the bio-safety level 3 plus facilities of the Virology Department, Ankara University of Turkey. 

### 2.2. Cells

Baby hamster kidney (BHK21-C13) and Scott and White No. 13 (SW-13) cells were used for the transfection and virus propagation assays, respectively. Cell maintenance was provided by Dulbecco’s Modified Eagle’s medium (DMEM) media supplemented by 10% fetal bovine serum (FBS), 2 mM of L-glutamine, 100 IU/mL of penicillin (Biological Industries, Israel), 100 µg/mL of streptomycin (Biological Industries, Israel), and 2.5 µg/mL of Amphotericin B. The cells were sub-cultured 1/4 once a week. In the immunological assays, splenocyte cells were aseptically collected from all the immunized C57BL/6 mice and red blood cells were lyzed by RBC lysis buffer (Biological Industries, Kibbutz Beit-Haemek, Israel). After 2 wash steps with 1x phosphate buffered saline (1x PBS), the cell pellets were resuspended in RPMI-1640 medium (Sigma, St. Louis, MO, USA).

### 2.3. mRNA Vaccine Construct 

The pCD-N1 vector, previously created by insertion of the S segment of the Ank-2 strain of CCHFV into pCDNA3.1 myc/His A vector (Invitrogen, USA) [8], was used for PCR amplification of the small (S) segment. The amplified S segment was then exploited as a linear double-stranded DNA template for the mRNA vaccine creation. To perform the reaction, a primer set of F: 5′**-TAATACGACTCACTATAGGGAGA***GGGAAATAAGAGAGAAAAGAAGAGTAAGAAGAAATATAAGAGCCACC*ATGGAAAACAAGATCGAGGT-3′ and 

R: 5′-CCCGCTGGGCCTCCCAACGGGCCCTCCTCCCCTCCTTGCACC

TTAGATGATGTTGGCACTGG-3′ was designed. The PCR reaction was carried out by Phusion High Fidelity Taq DNA polymerase (Thermo Fisher Scientific, Waltham, MA, USA) from pCD-N1 as follows: initial denaturation at 98 °C for 30 s, and 30 cycles of denaturation at 98 °C for 10 s, annealing at 55 °C for 20 s and extension at 72 °C for 40 s, followed by final extension at 72 °C for 5 min. The mRNAs were created from the gel-purified PCR products by mMESSAGE mMACHINE™ T7 ULTRA Transcription Kit (Thermo Fisher Scientific, Waltham, MA, USA) according to the manufacturer′s instruction. This kit uses the T7 promoter as the initiation site of transcription. We therefore included this promoter (bold letters) in the forward primer. In addition, the 5′-optimized untranslated region (UTR; Italic letters) was added to this primer, as described elsewhere [24]. The reverse primer included α-globin 3′-UTR (italic letters). Both UTRs were added to guarantee the stability of the mRNA construct [25]. To create the mRNA from the PCR product, the reaction was assembled as follows: 100 ng of purified PCR product, 10 µL of T7 2X NTP/ARCA, 2 µL of 10X T7 reaction buffer, 2 µL of T7 enzyme and nuclease-free water in a total of 20 µL. The mix was incubated at 37 °C for 2 h. This kit provides a 5′-cap by using 5′-Anti-Reverse Cap Analog (ARCA) and 50–100 poly (A)-tail in addition to the in vitro transcription. Finally, the construct was verified by running in denaturing agarose-formaldehyde gel, according to the mRNA synthesis kit’s instructions, and purified using MEGAclear™ Kit, according to the manufacturer’s instructions.

### 2.4. pET-N13 Construct and Western Blot Assay

Total RNAs were extracted from the CCHFV Ank-2 strain infected SW-13 cells by using TRIzol™ reagent (Thermo Fisher Scientific, Waltham, MA, USA) according to the standard protocol [26]. After cDNA synthesis by using SuperScript III Reverse Transcriptase (Thermo Fisher Scientific, Waltham, MA, USA), the PCR product was amplified by a primer set of F: GAAAACAAGATCGAGCT and R: GATGATGTTGGCACTGG. The reaction was set up as follows: initial denaturation at 98 °C for 30 s, and 35 cycles of denaturation at 98 °C for 10 s, annealing at 52 °C for 20 s and extension at 72 °C for 40 s, followed by a final extension at 72 °C for 5 min. The PCR product contained the complete small segment of the Ank-2 strain without start and stop codon to guarantee fusion of the His-tag during blunt cloning of the insert into the pET-28a vector. After verification of the constructs by sequencing, we induced the his-tagged nucleocapsid protein expression in BL21 bacteria (NEB, Ipswich, MA, USA) using 0.5 mM Isopropyl β-D-1-thiogalactopyranoside (IPTG; Sigma, St. Louis, MO, USA) at 16 °C for 20 h [27]. The his-tagged protein was then purified with a MagneHis™ Protein Purification System (Promega, Madison, WI, USA) and verified by western blot assay to detect the 52 kDa protein of interest plus 5 kDa double His6-Tag on the membrane as previously described [8]. Briefly, the collected His-tagged nucleocapsid proteins were denatured at 95 °C for 5 min and separated in Mini-Protean TGX Stain-Free precast gels (BioRad, Hercules, CA, USA) in 1xTris/Glycine/SDS buffer for 2 h at 100 V. They were then transferred to a polyvinylidene difluoride (PVDF; BioRad, Hercules, CA, USA) membrane using the Trans-Blot Turbo Transfer System (BioRad, Hercules, CA, USA). A 1/250 dilution of human polyclonal anti-CCHFV IgG was added and the membrane was incubated at room temperature (RT) for 90 min followed by incubation in the presence of anti-human IgG-HRP secondary antibody (Sigma, St. Louis, MO, USA) at a dilution of 1/750 for 1 h at RT. Finally, they were visualized by adding Clarity Western ECL substrate solution (BioRad, Hercules, CA, USA) in the ChemiDoc MP System (BioRad, Hercules, CA, USA).

### 2.5. Indirect Immunofluorescence Assay (IIFA)

The mRNA construct was transfected into the BHK21-C13 cells using Lipofectamine3000 (Thermo Fisher Scientific, Waltham, MA, USA) according to the manufacturer′s instruction. After 72 h, the transfected cells were fixed with 3.7% formaldehyde and permeabilized in the presence of 0.1% triton-X100. Following a blocking step using 5% skimmed milk buffer, human polyclonal anti-CCHFV antibody at a dilution of 1/250 was added and incubated at 4 °C overnight. The next day, the cells were incubated in the presence of anti-human IgG-FITC secondary antibody (Sigma, St. Louis, MO, USA) at 1/1000 dilution for 1 hour at RT. They were then visualized by Axio Vert A1 Microscope (Ziess, Oberkochen, Germany). As a positive control, we used siRNA (TX-Red) (System BioSciences, Palo Alto, CA, USA) transfection.

### 2.6. Immunization Schedule in C57BL/6 Mice

To analyze the immunogenicity of the mRNA construct, we immunized immunocompetent C57BL/6 mice as follows. In this experiment, a total of 12 female C57BL/6 mice (8–12 weeks) were randomly divided into 3 groups of single, booster dose, and negative control (normal saline). In the single dose group, each mouse received 25 µg of mRNA construct eluted in 100 µL of normal saline buffer and injected through the thigh muscle of the hind limb (i.m.). In the booster dose group, the mice received an additional dose of the same regime and injection route, two weeks after the first injection. The control group received 100 µL of normal saline through the same i.m. route. All the immunized mice were observed daily to document the onset of any allergic signs. Blood samples were collected from the tail vein based on our laboratory’s routine every 2 weeks and the collected serums were stored at −80 °C until further use in the sandwich in-house ELISA and virus neutralization assays. The immunized mice were humanely euthanized 4 weeks after the final injection to collect splenocyte cells for intracellular cytokine staining and ELISPOT assays.

### 2.7. Challenge Virus and Experiment

The challenge experiment was performed in the IFNα/β/γR^−/−^ mice model using 100LD_50_ (1000TCID_50_) of the third passage of the Ank-2 strain (access number: MK309333) of CCHFV in SW-13 cells as previously described [5]. Briefly, 12 8–10-week-old healthy female mice were randomly divided into 3 groups of single, booster, and negative control. Each group received the same dose of mRNA construct on the routine schedule as previously described for the C57BL/6 mice. Four weeks after the final injection of the vaccine, each mouse was inoculated with 300 µL of the challenging virus through the intraperitoneal route (i.p.). The infected mice were daily observed to document the onset of clinical symptoms, such as appearance changes (nasal and ocular discharges), depression, bodyweight loss/gain, paralysis, and death. We selected body weight percentage as an indicator of the end of the challenge assay. Once the animals had gained the challenged day’s body weight with no significant clinical signs, we ended the experiment. Besides, mice showing more than 20% of body weight loss were considered to have reached the experimental endpoint and were humanely euthanized. Under these conditions, the challenge experiment continued for 25 days, after which all the animals were humanely euthanized. 

### 2.8. IFN-Gamma and IL-4 ELISPOT Assays in C57BL/C Mice

The ELISPOT assays were performed by ELISPOT Mouse IFN-gamma and IL-4 kits (R&D Systems, Minneapolis, MN, USA) based on the established protocol of the manufacturer. Briefly, splenocyte cells from each group of immunized C57BL/6 mice (4 weeks after the mRNA vaccine injection in single dose group and the same period after the booster injection in the booster group) were pooled and 3 × 10^5^ cells were added onto each well of 96 well-plates in triplicate. Ten micrograms of the nucleocapsid protein purified from BL-21 bacteria was added to each well to stimulate the production of the cytokines for 6 h and then 100 µL of each of diluted detection antibody was added and incubated at +4 °C overnight. In the next step, 100 µL of the diluted Streptavidin-AP antibody was added for 2 h at RT to each well and finally, by addition of BCIP/NTB chromogen for 1 hour at RT, the assays were terminated and the IFN-gamma and IL-4 specific spots were counted by a dissection microscope. As a negative control, we included the naïve splenocyte cells from unvaccinated mice in the experiment.

### 2.9. In-House Sandwich ELISA (C57BL/6 and IFNα/β/γR^−/−^ Mice)

The anti-nucleocapsid IgG1 and IgG2a antibodies in the sera of immunized C57BL/6 and IFNα/β/γR^−/−^ mice (on days 0, 14, 28, and 42 for the single dose group and days 0, 14, 28, and 56 for the booster dose group) were assessed through the in-house sandwich ELISA assay, developed as previously described [20]. Briefly, SW-13 cells were mock-infected or infected with 1 *moi* of Ank-2 and collected 5 days post-inoculation when the CPEs reached about 90% of the cells. After 3 rounds of freeze-thaw and 1 round of sonication, the cell lysates were coated on a 96-well MaxiSorp ELISA plate (Thermo Fisher Scientific, Waltham, MA, USA) for 24 h at +4 °C in the presence of carbonate/bicarbonate buffer, pH 9.6. After fixation with 1% formaldehyde (20 minutes at RT) and permeabilization with 0.1% triton-X (15 minutes at RT), the serum samples were serially diluted in RPMI-1640 medium (Thermo Fisher Scientific, Waltham, MA, USA), added onto the wells in duplicate and incubated at 4 °C overnight. The next day, after 3 rounds of wash, biotinylated-anti-mouse IgG1 and biotinylated-anti-mouse IgG2a (BioLegend, San Diego, CA, USA) at 1/1000 dilution were separately added to each well. After 2 h of incubation at RT, HRP-streptavidin (BioLegend, San Diego, CA, USA) at a dilution of 1/1000 was added for 1 hour at RT followed by 3, 3′, 5, 5′-Tetramethylbenzidine (TMB ELISA Peroxidase) substrate color development. The reaction was stopped by 2N H_2_SO_4_. The assay was read at 450 nm in an ELISA reader (Titertek Multiskan PLUS MK II Microplate Reader, Midland, ON, Canada). The results of the unvaccinated mice serum samples were subtracted from the cell lysate as background.

### 2.10. Virus Neutralization Assay (C57BL/6 and IFNα/β/γR^−/−^ Mice)

The serum samples collected on days 0, 14, 28, and 42 for the single dose group and days 0, 14, 28, and 56 for the booster dose group from C57BL/6 and IFNα/β/γR^−/−^ mice were subjected to virus neutralization assay. Briefly, the serums were inactivated at 56 °C for 30 min and then, an equal volume of 1/2 diluted serum was mixed with the Ank-2 strain of CCHFV at 100TCID_50_ titer followed by incubation at 37 °C for 1 hour. Each mixture was added to the SW-13 cells in 96-well plates in four wells. As a positive control, we included the virus infected cells. The cells were observed daily for the onset of cytopathic effects (CPEs) including the rounded and detached cells. The assay was terminated on day 5 post-inoculation when the CPEs reached 90% in the positive control wells. 

### 2.11. Intracellular IFN-Gamma Detection in Antigen-Activated CD8a+ and CD4+ T Cells (C57BL/6 Mice) 

Splenocyte cells from the immunized C57BL/6 mice (on day 42 for the single dose group and day 56 for the booster dose group) were aseptically collected, and the CD4+ and CD8a+ cells were separated by MojoSort™ Mouse CD4 and CD8a Nanobeads (BioLegend, San Diego, CA, USA) according to the manufacturer’s instructions. Briefly, the splenocyte cells (10^7^ cells) were prepared in 100 µL of MojoSort™ Buffer (BioLegend, San Diego, CA, USA) before 5 µL of rat serum (Sigma, St. Louis, MO, USA) was added and incubated for 10 minutes at RT. In the next step, after the addition of 10µL of Antibody Nanobeads (15 min on ice) and 2 rounds of wash by MojoSort™ Buffer, the CD4+ and CD8a+ positive cells were collected in a total of 3 separations using the magnet. Before proceeding to the next step, the CD4+ and CD8a+ cells were verified with PE-anti-mouse CD4+ and CD8a+ antibodies (BioLegend, San Diego, CA, USA) in cytoFlex cytometry (Beckman Coulter, Atlanta, GA, USA). To determine the intracellular IFN-gamma response in the collected CD4+ and CD8a+ cells, we added 20 µg of nucleocapsid protein and 1x brefeldin A (BioLegend, San Diego, CA, USA) to the 1x10^6^ cells for 6 h at 37 °C to stimulate cytokine production and inhibit protein transportation, respectively. Fixation and permeabilization were performed using Fix & Perm / Cell Fixation & Permeabilization Kit (Abcam, Cambridge, MA, USA) according to the manufacturer’s instructions. Briefly, 100 µL of fixation reagent was added to 50 µL of the cell suspension in a 5 mL tube and incubated at RT for 15 min. Then, 5 mL of 1× PBS was added and centrifuged at 300 × *g* for 5 minutes. In the final step, the cell pellet was resuspended in the 100 µL of permeabilization reagent. Along with the second solution, a 1/1000 dilution of FITC-anti-mouse IFN-gamma antibody (BioLegend, San Diego, CA, USA) was added and incubated at RT for 20 min. The results were obtained by using CytoFlex cytometry and the data were analyzed using Cytexpert software (Beckman Coulter, Atlanta, GA, USA).

### 2.12. Statistical Analysis

The anti-nucleocapsid specific antibody isotyping data and the intracellular IFN-gamma cytokine levels among the groups were evaluated using a two-way (Sidak’s post hoc correction) ANOVA by GraphPad Prism version 7.0 (GraphPad Software, San Diego, CA, USA; www.graphpad.com). All graphs were created by the same program. Data were considered statistically significant when *p* < 0.05. Due to the restricted resources, the optimal sample size based on the power was assessed by using the online tools available at http://www.sample-size.net/means-effect-size/ performed before the experiment and based on the power rate obtained in this step, the validity of the results was verified by an additional online tool (http://hedwig.mgh.harvard.edu/sample_size/size.html) in which the true difference between treatments is 1.798 times the standard deviation.

## 3. Results

### 3.1. mRNA Vaccine Construction and In Vitro Expression

**Figure 1:**
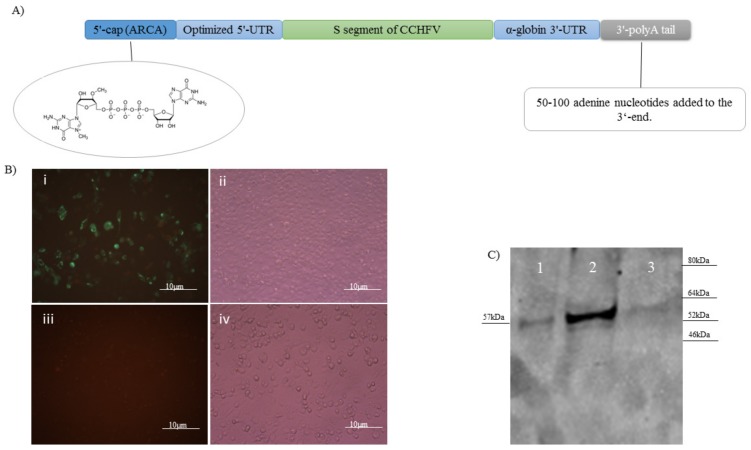
(**A**) Genomic structure of naked conventional mRNA expressing the non-optimized S segment of the Ank-2 strain of CCHFV. To guarantee the stability of the structure, a 5′-cap, 3′-poly A tail, and 5′ and 3′-UTR were added. (**B**) The created mRNA vaccine construct was transfected in the BHK21-C13 cells and in vitro expression was verified by IIFA (×40): (**i**) fluorescence; (**ii**) phase contrast. We also included negative control cells as the background in the assay: (**iii**) fluorescence; (**iv**) phase contrast. (**C**) Expression of the His-tagged nucleocapsid protein in the bacterial system was analyzed by western blot assay. As became obvious after overnight IPTG induction of BL21 bacteria containing pET-N13, a strong band of 57 kDa (lane 2) was detected in the WB. As a control, we included the supernatant from the IPTG induced (lane 1) and the lysate from the non-induced bacteria containing the pET-N13 construct (lane 3).

The mRNA construct expressing the nucleocapsid protein was developed using a commercial in vitro transcription kit. In addition to the mRNA synthesis, this kit provided the 5′- cap (ARCA) and 50-100 bp 3′-polyA tail in the construct’s structure to guarantee in vivo stability. The 3′ and 5′-UTRs were added to the primers used to amplify the S segment. This PCR product was used as the template for mRNA construction. Thus, the final mRNA construct, as the vaccine candidate, contained the 3′ and 5′-UTRs, 5′- cap (ARCA), and 3′-polyA tail (Figure 1A). The final construct was verified by running in agarose-formaldehyde gel to detect the right size. In addition, the presence of the polyA tail in the final construct was also confirmed in the gel as described by the kit. To verify in vitro expression, we conducted IIFA in the BHK21-C13 cells analyzed on day 3 post-transfection. The construct had a significant expression level in vitro as detected by the presence of green spots (Figure 1B).

### 3.2. Western Blot Assay

To analyze the purified His-tagged nucleocapsid protein expressed from the pET-N13 construct in the induced BL21 bacteria, we ran the purified proteins obtained from the bacterial lysates in 4–12% gradient SDS gel and detected the right size of 57 kDa (52 kDa of the nucleocapsid plus 5 kDa of the his-tag) on the gel (Figure 1C; lane 2). No detectable non-specific or extra bands were observed in the assay. As a control, we included the supernatant from the IPTG induced and the lysate from the non-induced bacteria containing the pET-N13 plasmid in the assay. We observed a weak specific band in the supernatant of the induced bacteria, which indicates that the expressed protein was also present in the supernatant (Figure 1C; lane 1).

### 3.3. Anti-Nucleocapsid IgG1, and IgG2a Antibody Responses (C57BL/6 and IFNα/β/γR^−/−^Mice)

**Figure 2:**
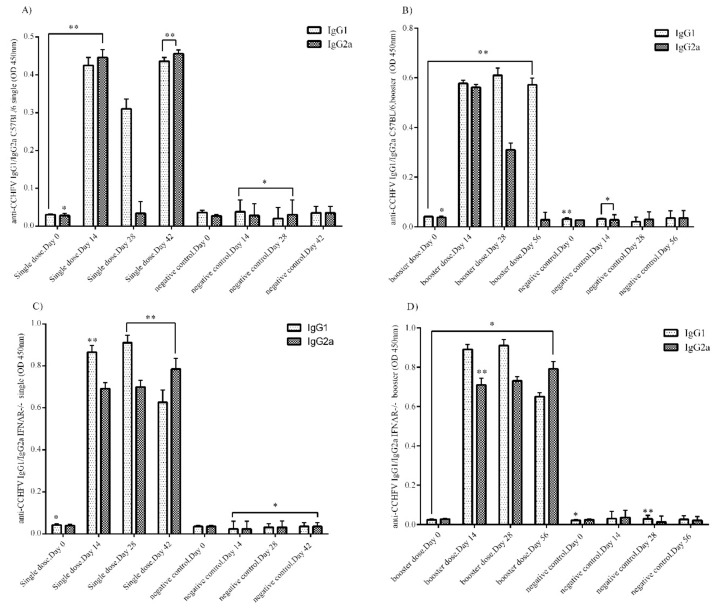
In-house sandwich ELISA assay. (**A**) Anti-nucleocapsid specific IgG1 and IgG2a responses in the serum samples of C57BL/6 mice in the single dose group. IgG1 and IgG2a responses in the single dose group peaked on days 14 and 42 (p.i). IgG2a levels were low on day 28 p.i. (**B**) Anti- nucleocapsid specific IgG1 and IgG2a responses in the serum samples of C57BL/6 mice in the booster dose group. IgG1 response was higher on days 14, 28, and 42 p.i., whereas IgG2a levels were higher on day 14 p.i but lower on days 28 and 42 p.i. (**C**) Anti-nucleocapsid specific IgG1 and IgG2a responses in the serum samples of IFNα/β/γR^−/−^ mice in the single dose group. Both IgG1 and IgG2a levels were higher on days 14, 28, and 56 p.i. IgG1 responses were higher than IgG2a. (**D**) Anti- nucleocapsid specific IgG1 and IgG2a responses in the serum samples of IFNα/β/γR^−/−^ mice in the booster dose group. Levels were the same as for the IFNα/β/γR^−/−^ single dose group. IgG1 and IgG2a responses were both high. All ELISA assay data were obtained from serums at 1/100 dilution, analyzed using the log-rank test (* *P* < 0.05; ** *P* < 0.01; and *** *P* < 0.001), and presented as mean ± sd. Error bars indicate standard deviations (SD).

In the 1/100 diluted serum samples of the immunized C57BL/6 and IFNα/β/γR^−/−^ mice, the amount of anti-nucleocapsid IgG1, and IgG2a antibodies on days 0, 14, 28, and 42 for the single dose group and days 0, 14, 28, and 56 for the booster dose group were analyzed by in-house sandwich ELISA. The IgG1 responses in the single dose group of the immunized C57BL/6 mice were relatively high. In addition, IgG2a antibody reached a significant level on days 14 and 42 post-immunization (p.i.). Surprisingly, this antibody had a low level on day 28 p.i. (Figure 2A). In the booster group of C57BL/6 mice, IgG1 levels were higher than IgG2a. On days 14, 28, and 56 p.i., IgG1 had a high level whereas IgG2a antibody levels dramatically dropped from day 14 to day 56 p.i. (Figure 2B). The results for both antibodies in both groups of C57BL/6 mice demonstrated that the booster dose injection elicited more specific antibody responses than the single dose administration. 

Regarding the IgG1 and IgG2a responses in the single dose group of the immunized IFNα/β/γR^−/−^ mice, both antibodies were detected at high levels in the serums (Figure 2C). In this group, there were higher levels of IgG1 than IgG2a on days 14 and 28. On the other hand, IgG2a was higher on day 42 p.i. The highest antibody responses in the single dose group were for IgG1 on day 28 p.i. The results for the booster dose group of IFNα/β/γR^−/−^ mice were similar to those of the single dose group. Both IgG1 and IgG2a antibody production was higher than in the negative control group. Like the single dose group, IgG1 levels were higher than IgG2a on days 14 and 28 p.i. (Figure 2D). Comparing IgG1 and IgG2a levels in the single and booster dose groups at the same timepoints, the antibody levels were not different. This indicates that the booster dose does not stimulate more production of either antibody than that induced by the single dose injection. 

### 3.4. Virus Neutralization Assay

None of the serum samples obtained from both the vaccine and normal saline immunized C57BL/6 and IFNα/β/γR^−/−^ mice on days 0, 14, 28, and 42 for the single dose group and days 0, 14, 28, and 56 for the booster dose group p.i. could neutralize the Ank-2 strain of CCHFV in vitro.

### 3.5. ELISPOT Assays (C57BL/6 Mice)

**Figure 3:**
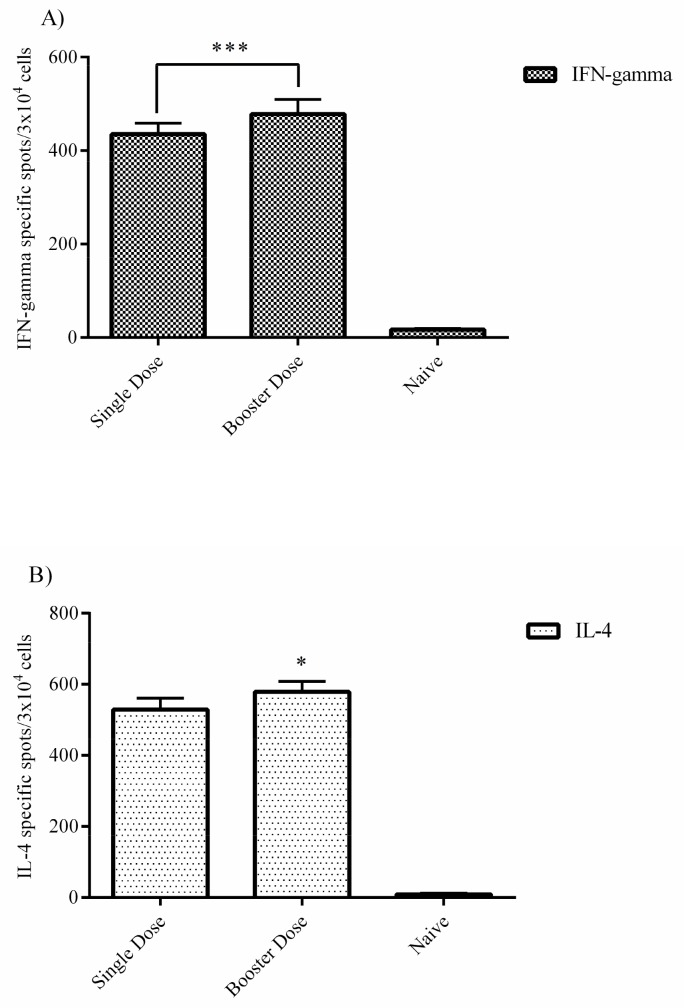
ELISPOT assays. (**A**) Splenocyte cells from immunized C57BL/6 were obtained on week four after immunization. IFN-gamma secretion was analyzed by ELISPOT assay. The number of IFN-gamma specific spots in the booster dose group was higher than for the single dose. (**B**) The number of IL-4-specific spots was similar to that for IFN-gamma. This indicates that the booster dose had a stronger effect than the single dose. The data was analyzed using the log-rank test (* *P* < 0.05; ** *P* < 0.01; and *** *P* < 0.001) and presented as mean ± SD. Error bars indicate standard deviations (SD).

As an indicator of Th1 and Th2 responses, we conducted IFN-gamma and IL-4 ELISPOT assays in the splenocyte cells obtained from immunized C57BL/6 mice on day 42 for the single dose group and day 56 for the booster dose group p.i. The IFN-gamma-specific spots detected in the splenocyte cells of the single dose group are significant and higher in the booster dose group, which indicates a dose-dependent increase in production of this cytokine (Figure 3A). The results for IL-4 were the same as those for IFN-gamma (Figure 3B). More IL-4 specific spots were counted in the booster dose group than in the single dose group, and the same dose-dependent phenomenon was also observed in this assay. Our data demonstrate that the Th2 response (obtained from IL-4) is stronger than that of Th2 (obtained from IFN-gamma). As a negative control, we included naive splenocyte cells from normal saline immunized negative control mice.

### 3.6. Intracellular IFN-Gamma Staining (C57BL/6 Mice)

**Figure 4:**
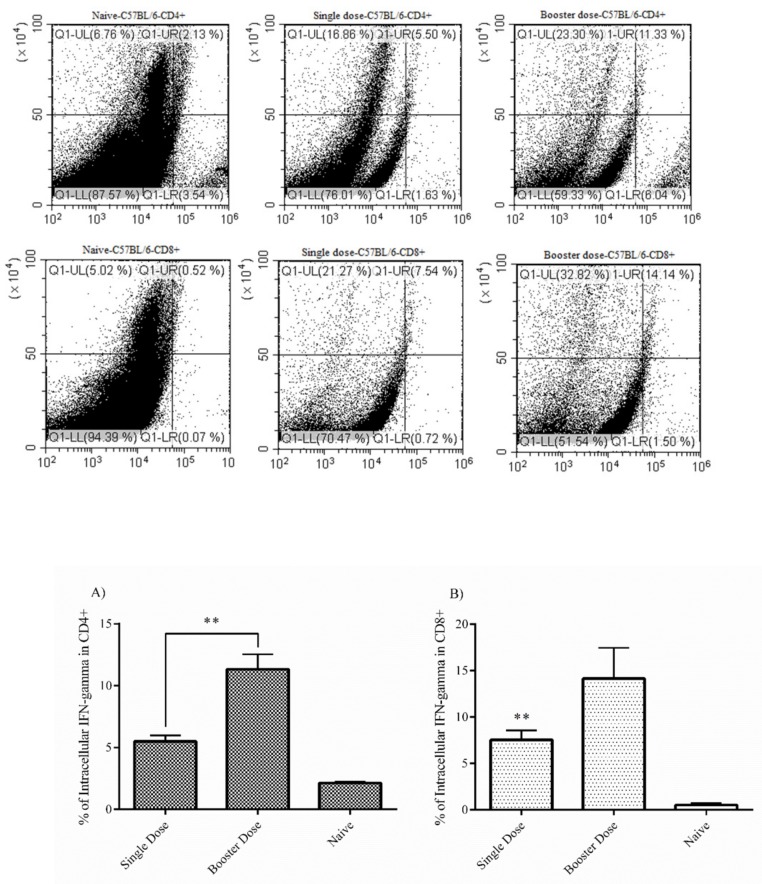
Intracellular IFN-gamma staining. CD4+ and CD8a+ T cells from immunized C57BL/6 mice (4 weeks after the final injection of mRNA construct) were separated in vitro and intracellular IFN-gamma staining was separately performed in each cell population. (**A**) Based on the flow cytometry analysis, the booster dose group clearly has more potential to stimulate the production of IFN-gamma cytokine in the CD4+ T cell population. (**B**) The results for the CD8a+ cells are similar to those for the CD4+ cell population, indicating that the booster dose has a stronger effect than the single dose. As a control, we used naive splenocyte cells from the negative control group. The data was analyzed using the log-rank test (* *P* < 0.05; ** *P* < 0.01; and *** *P* < 0.001) and presented as mean ± SD. Error bars indicate standard deviation (SD).

The level of intracellular IFN-gamma responses in CD4+ cells was higher in the booster dose group (11.33%) than for the single dose group, although the single dose group’s response was also significant (5.50%). This indicates that both single and booster dose administration elicited production of this cytokine at a relatively high level in this cell population (Figure 4A). For the CD8a+ T cell population, the booster dose group’s response (14.14%) was stronger than that of the single dose group (7.54%), similar to CD4+ cells (Figure 4B). The data for both CD4+ and CD8a+ cells indicates that the booster dose injection elicited more cytokine responses than the single dose. In addition, this cytokine was produced at higher levels in the CD8a+ than CD4+ cells. 

### 3.7. Challenge Experiment (IFNα/β/γR^−/−^ Mice)

**Figure 5:**
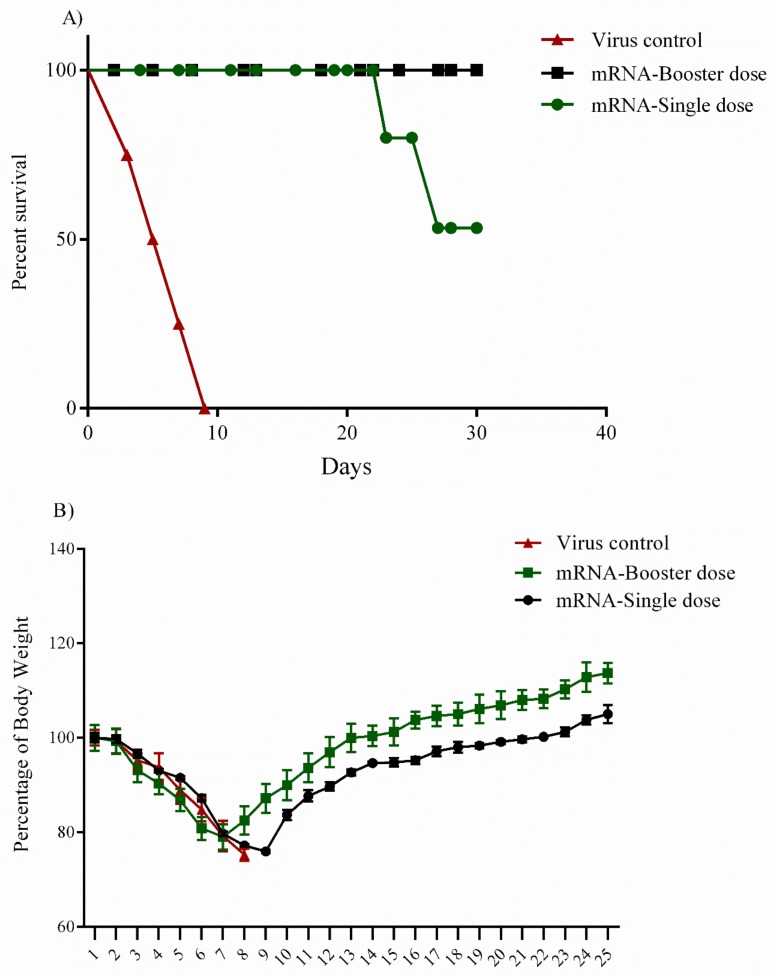
Challenge assay in IFNα/β/γR^−/−^ mice. (**A**) Percentage survival. A total of 12 female 8-12-week-old IFNα/β/γR^−/−^ mice in 3 groups of single, booster, and negative control were immunized with conventional mRNA construct. After 4 weeks p.i., each mouse received 100LD_50_ of the Ank-2 strain of CCHFV at a final volume of 300 µL and was monitored daily for the onset of clinical symptoms. The protection rate was 100% for mice in the booster dose group in the challenge assay whereas the protection rate was 50% in the single dose group. (**B**) Percentage of initial body weight. Percentage change in body weight was used as an indicator of the challenged mice’s health condition. The mice in the booster dose group had a clearly faster recovery rate than the mice in the single dose group. The mice in the booster dose group also gained more weight than the single group by the end of the experiment on day 25 post-challenge. Regarding the positive control, the mice in the virus group died on days 3, 5, 7, and 9 post-challenge. All the data are presented as mean±sd. Error bars indicate standard deviation (SD).

A total of 12 female 8–12 week-old IFNα/β/γR^−/−^ mice in 3 groups of single, booster and negative control were immunized by the mRNA construct. Protection rates of 100% and 50% were noted in booster and single dose groups, respectively (Figure 5A). In the single dose group, the infected animals started to develop clinical symptoms including nasal and ocular discharge, depression, disheveled appearance and, in some cases, paralysis in the lower limbs during the 3 days post-injection. The symptoms started to disappear 8–10 days following challenge in the survived animals. Interestingly, one mouse in the single dose group showed an unusual pattern of unilateral paralysis, resulting in death 2 days after the onset of clinical signs. It is worth mentioning that the CCHFV Ank-2 strain demonstrated some neurotrophic characteristics in our previous studies [5,8]. In the single dose group, 2 cases of death occurred on days 23 and 27 post-inoculation. Weight loss and/or gain were observed as an indicator of progression and experiment termination. It was observed that the booster group started to recover faster in comparison to the single dose group. On the other hand, the booster dose group showed a lower amount of the body weight loss (23%) after the challenge experiment. The average weight loss of the single dose group was almost 26%. In infected mice, weight loss started from day 1 post-inoculation and continued until day 10, as the survivors gradually gained weight afterward. The virus controls animals perished within 8 days following inoculation (Figure 5B).

## 4. Discussion

Crimean-Congo Hemorrhagic Fever (CCHF) is becoming a major health concern in endemic areas like Turkey. Thus, finding effective therapeutics or vaccination approaches is critical [3]. Vaccine design based on delivering two main immunodominant viral genes (small and medium) has had positive immunological results, indicating their potential to be considered as the main vaccine targets [28].

There are two main types of obstacles which researchers encounter in the field of CCHFV vaccine design. Firstly, the challenge experiments must be performed in the genetically modified models, such as the immunocompromised signal transducer and activator of transcription 1 knock-out (STAT1−/−) [29], interferon α/β receptor knock-out (IFNα/β/γR^−/−^) [30], or interferon receptor antibody transiently suppressed (IS) mouse models, due to the susceptibility of this virus to interferon cytokines [31]. Thus, the results cannot be considered as valid to further enter the human trials. Recently, new generations of laboratory animals, called humanized mice, have been introduced to make such results more rational [32]. Although immunocompetent mice models are generally not suitable for CCHFV vaccine study [31], the humoral responses obtained from this animal model may be useful for CCHFV pathology research. For instance, one study demonstrated that more balanced humoral (IgG2c/IgG1) responses were detected in immunocompetent mice (C57BL/6) than for IFNAR^−/−^, which may be due to cytokine signaling differences in the immunocompetent mice [33]. In this regard, the present study produced several important findings. Both mouse models elicited the significant nucleocapsid-specific IgG1 and IgG2a responses. Levels of both antibodies in the IFNα/β/γR^−/−^ mice were higher than the C57BL/6 when compared at the same time point post infection (p.i). In addition, the booster dose injection in C57BL/6 showed higher responses of both antibodies than the single dose group. On the other hand, there were no significant differences in antibody levels from both the single and booster doses of IFNα/β/γR^−/−^ mice. The data obtained from IFNα/β/γR^−/−^ mice showed that the sole presence of the specific antibody responses is not sufficient for eliciting the protection in the challenge assay. This finding may be worth exploring in future experiments, as it suggests that the antibody response in immunocompetent mice may be more reliable than the interferon knock-out mice. 

The second hindrance regarding the vaccine trials is that researchers may have to develop new strategies to combat different viral diseases including CCHFV. This is because of various drawbacks of traditional vaccines, such as the high cost of the cell culture systems used for pathogen propagation, low vaccine yields, ineffectiveness of vaccine constructs due to pre-existing immunity, and the emergence of new pathogens by mechanisms such as recombination or reassortment in the viruses [34]. Additionally, vaccine development against some pathogens like HIV-1 was almost unsuccessful using traditional approaches [35]. Given these circumstances, one of the main considerations in CCHFV vaccine design is the selection of the suitable expression platform. For instance, a prime and booster regime of a modified Ankara vaccinia virus (MVA) encoding the M segment (GPC) has provided a protection rate of 100% in IFNα/β/γR^−/−^ mice [9]. On the other hand, the same expression platform and regime when delivered the S segment failed to elicit sufficient immune response to protect the mouse model [36].

To overcome these obstacles, new vaccine platforms such as virus-like particles, cellular and conjugate vaccines, and nucleic acids (DNA and mRNA) have been introduced [37]. Of these, nucleic acid gene transfer has attracted great interest because of its obvious advantages, such as easy propagation and manipulation, no risk of infection, construct stability for shipment, long-term immunogenicity and polarization of T cells towards Th1 and Th2 [38]. However, DNA vaccines also carry risks that hinder their use in clinical trials. Specifically, these constructs stimulate the immune cells to produce antibodies against the transfer backbone and show relatively poor immunogenicity when administrated alone. Finally, some experiments have demonstrated that these constructs may integrate themselves into the host genome [39].

The mRNA construct was first used as a new expression platform using the mouse model by injecting a construct containing chloramphenicol acetyl transferase (CAT) coding sequences flanked by α-globin 5′ and 3′ untranslated sequences and a 3′ polyadenylate tract. CAT activity was detected in all mRNA vaccinated mice [40]. During the last decade, there has been a huge investment in mRNA vaccine development due to its obvious advantages over other expression platforms [41,42].

To the best of our knowledge, our study is the first to develop a naked conventional mRNA construct expressing the small segment of CCHFV. The mRNA construct is safe, non-immunogenic, and non-integrated. Although it has a short half-life in vivo, this can be extended by modifications to obtain the desired results during the vaccination [18]. Furthermore, large quantities of mRNA vaccine can be produced by cell-free enzymatic transcription reactions, which can be considered as a distinguishing factor in this field [43].

One of the main factors when developing new mRNA vaccines is codon optimization of the gene of interest by substitution of the rare amino acids’ codons to guarantee high-level protein expression during in vitro and in vivo experiments [44]. In the present study, we used the wild type (non-optimized) S segment of the Ank-2 strain of CCHFV as our template and this fact did not clearly affect our immunological results as they were detected at a significant level in both animal models. Both the single and booster doses of this non-optimized construct stimulated immune responses and protected IFNα/β/γR^−/−^ mice in the challenge assay. Nevertheless, it is important for future studies to analyze the effect of codon optimization of the S segment of CCHFV in the immune response for enhancement. The importance of this can be understood from our challenge assay results, which demonstrated a protection rate of 100% in the booster group but only 50% in the single dose group. Besides, our construct incorporated some codon optimization, specifically 5′ capping, 5′ and 3′ UTRs, and a polyA tail, to increase in vivo half-life of the construct. 

Another interesting aspect of mRNA constructs is their potential to elicit dendritic cell maturation, which promotes robust T and B cell responses [45]. This characteristic was seen in our study from the results of the in-house ELISA and IFN-gamma responses. Our construct produced significant levels of anti-nucleocapsid specific IgG1 and IgG2a antibodies and interferon-gamma cytokines in both the single and booster dose groups. Despite the lack of neutralizing antibodies, both humoral and cellular responses are necessary to elicit protection in the challenge assay. Therefore, we conferred that the high amount of IFN-gamma responses and elevated levels of the specific IgG1 and IgG2a antibodies which were documented in the booster dose group, elicited full protection in the challenged IFNα/β/γR^−/−^ mice. However, the results of the interferon cytokine in the single dose group can justify the low rate of the protection in this group. This low immunogenicity might have originated from the fact that our construct is delivered as naked, and we know that this kind of construct has a short half-life in vivo. The results of the single dose group can be improved by arming the naked construct (obtains by using protamine, cationic lipid, and polymer-based delivery) in future experiments. Based on the immunological data obtained in this study and our previous study of CCHFV, in which we conducted passive antibody and T cell transfer assays in the mouse model, we demonstrated that these two arms of the immune response are complementary to elicit protection in genetically modified mouse model (IFNα/β/γR^−/−^) [8,9].

The level of protein expression from the mRNA construct is also influenced by its purity [21]. In the present experiment, we used a commercial kit to purify the final construct. For large scale production and Good Manufacturing Practice (GMP) processes, technologies like protein liquid chromatography (FPLC) or high-performance liquid chromatography (HPLC) may help increase purity and guarantee high expression levels of mRNA constructs in vivo [46].

The route of injection is also critical in mRNA vaccine administration. For mRNA, diverse studies have shown that intra-muscular and intra-dermal administration may produce higher expression levels than systemic delivery routes [47,48,49]. For instance, intranodal injection of a naked mRNA vaccine against the influenza virus elicited robust antigen-specific T cell responses in the mouse model whereas subcutaneous administration produced no immune responses [50]. We used intramuscular administration of the naked conventional mRNA construct and had mostly good immunological results. This supports a previous study reporting high immune responses in rodents after intramuscular injection of buffer-formulated naked mRNA [51]. However, because CCHFV is naturally transmitted by tick bite, it will be logical to try intra-dermal administration of our vaccine construct to examine immune response at the natural sites of virus entrance [52].

Our immunological results indicate that we have successfully introduced a new platform for CCHFV vaccination, which can be considered as a potential rival for conventional expression platforms. Our construct achieved a protection rate of 100% after the booster dose. The main consideration in our study is the onset of clinical signs in all immunized animals, including the single and booster dose groups. More specifically, 8–10 days after the onset of the clinical symptoms, all animals in the booster dose group and 2 mice from the single dose group had recovered. The deaths of two mice in the single dose group occurred on days 23 and 27 post-challenge, which may have been caused by organ failure due to virus replication. In our previous study, which used adenoviral type 5 and Bovine herpesvirus type 4 vectored CCHFV candidates to deliver the small segment; the immunized mice showed no signs of disease after the challenge assay [8]. Several factors may explain this finding, such as suboptimal purity and codon optimization, which stopped the construct reaching its protein expression peak in vivo, as mentioned earlier. Besides, in the present study, we tried to introduce a local vaccine for CCHFV, which can be used in Turkey, or other neighboring areas like Iran, which ahve genetically similar viruses. The main limitation of this kind of study is the lack of challenge experiments with strains from different areas to establish a global vaccine. This should be considered in future studies.

In conclusion, the mRNA vaccine platform shows promising potential in the struggle against CCHFV infection and therefore deserves more attention due to its unique characteristics. However, the need for the platform optimization steps such as mRNA arming, purity increasing, and codon substitutions seem to be urging. Besides, we have demonstrated that the small segment of this virus can be considered as the main target for vaccine development because it provided a protection rate of 100% during the challenge assays in the IFNα/β/γR^−/−^ mice model. Further experiments are necessary to increase our knowledge about the T and B cell epitopes on the S segment in order to accurately design new, lower risk vaccines against this lethal virus.
